# Initial chest X-ray findings in pediatric patients diagnosed with H1N1 virus infection

**DOI:** 10.1590/0100-3984.2018.0030

**Published:** 2019

**Authors:** Isa Félix Adôrno, Tiago Kojun Tibana, Rômulo Florêncio Tristão Santos, Victor Machado Mendes Leão, Yvone Maia Brustoloni, Pedro Augusto Ignácio Silva, Marco Antônio Ferreira, Thiago Franchi Nunes

**Affiliations:** 1 Hospital Universitário Maria Aparecida Pedrossian da Universidade Federal de Mato Grosso do Sul (HUMAP-UFMS), Campo Grande, MS, Brazil.

**Keywords:** Influenza, human, Influenza A virus, H1N1 subtype, Radiography, thoracic, Influenza humana, Vírus da influenza A subtipo H1N1, Radiografia torácica

## Abstract

**Objective:**

To evaluate chest X-ray findings in pediatric patients diagnosed with influenza A (H1N1) virus infection.

**Materials and Methods:**

We retrospectively reviewed chest X-ray findings in 17 cases of pulmonary infection with the H1N1 virus (in 7 males and 10 females) examined between 2012 and 2016. The mean age of the patients was 14 months (range, 2-89 months). The diagnosis was established on the basis of clinical and radiographic criteria, and the virus was detected by polymerase chain reaction. The radiographic findings were categorized by type/pattern of opacity and by lung zone. The patients were divided into two groups: those not requiring ventilatory support; and those requiring ventilatory support or evolving to death.

**Results:**

The abnormality most often seen on chest X-rays was that of peribronchovascular opacities, the majority of which affected less than 25% of the lung, the involvement being bilateral and asymmetric. The lung zone most frequently involved was the middle third, with central and peripheral distribution, without pleural effusion. There was a statistically significant difference between the groups in terms of the symmetry of pulmonary involvement, asymmetric findings predominating in the group that required ventilatory support (*p* = 0.029).

**Conclusion:**

In pediatric patients with H1N1 virus infection, the main alterations on the initial chest X-rays are peribronchovascular opacities, nonspecific alveolar opacities, and consolidations. Although the definitive diagnosis of H1N1 virus infection cannot be made on the basis of imaging characteristics alone, using a combination of clinical and radiographic findings can substantially improve the diagnostic accuracy.

## Abstract (pt)

**Objetivo:**

Avaliar os achados na radiografia de tórax de pacientes com diagnóstico de infecção pelo vírus influenza.

**Materiais e Métodos:**

Revisamos, retrospectivamente, os achados na radiografia de tórax de 17 casos de infecção pulmonar pelo vírus influenza (7 do sexo masculino e 10 do sexo feminino; idade média de 14 meses, variação de 2 a 89 meses). Os pacientes foram examinados entre 2012 e 2016 e o diagnóstico foi estabelecido por critérios clinicorradiológicos e detecção do vírus por reação em cadeia de polimerase. Os achados radiológicos foram caracterizados por tipo e padrão de opacidade e distribuição por zonas pulmonares. A população estudada foi dividida em dois grupos: sem suporte ventilatório e com suporte ventilatório e/ou óbito.

**Resultados:**

A anormalidade encontrada com maior frequência na radiografia de tórax foram as marcas peribroncovasculares, a maioria delas com extensão menor de 25% do pulmão, envolvimento bilateral e assimétrico. A região mais frequentemente envolvida foi o terço médio, com distribuição central e periférica e ausência de derrame pleural. Houve diferença estatisticamente significante na simetria do envolvimento pulmonar, entre os grupos, havendo preponderância de achado assimétrico (*p* = 0,029) no grupo que necessitou de suporte ventilatório.

**Conclusão:**

Pacientes pediátricos com infecção pelo H1N1 apresentam como alterações principais na radiografia do tórax inicial marcas peribroncovasculares, opacidades alveolares inespecíficas e consolidações. Embora o diagnóstico definitivo não possa ser feito com base em imagens características isoladas, uma combinação dos achados clínicos e radiográficos pode melhorar substancialmente a acurácia do diagnóstico nessa doença.

## INTRODUCTION

In March of 2009, health authorities in Mexico reported a large outbreak of respiratory disease, which was later determined to be caused by infection with the swine-origin influenza A (H1N1) virus. Thirty days later, a case of infection with the H1N1 virus was detected in the United States. Within a few weeks, the virus had spread globally; on June 11, 2009, the World Health Organization declared H1N1 infection a global pandemic^(^^1^^,^^2^^)^.

For better management, the Brazilian National Ministry of Health divided the H1N1 pandemic into two phases^(^^3^^)^: containment and mitigation. The cases occurring in the containment phase were attributed to international travel or contact with infected individuals who had traveled abroad. In the mitigation phase, the National Ministry of Health recognized the occurrence of sustained person-to-person transmission within the country-a belated recognition, because there had already been deaths unrelated to transmission chains involving travelers^(^^4^^)^. In the central-west region, in the state of Mato Grosso do Sul, specifically in the greater metropolitan area of the city of Campo Grande, significant growth in the number of pediatric cases was observed beginning in 2012, the year we started collecting data related to patients with confirmed H1N1 infection.

Although several recent articles have described the computed tomography (CT) characteristics of H1N1 infection in adults^(^^1^^,^^5^^)^, there have been few population-based studies describing the early signs on chest X-ray as predictors of worse clinical outcomes in pediatric patients. We also found no studies comparing the patients who did and did not require invasive ventilation, in terms of the alterations observed. The main objective of this study was to characterize the initial radiographic patterns in pediatric patients with H1N1 influenza-associated pneumonia, and the secondary objective was to determine which radiographic signs are predictive of a worse clinical outcome.

## MATERIAL AND METHODS

The study was approved by the research ethics committee of our institution. Because of the observational nature of the study, the requirement for written informed consent was waived.

### Population studied

The data were collected from the medical records of the patients. The images were analyzed in the PACS of the diagnostic radiology department of our institution. We included all patients who were admitted to the pediatric ward of our hospital between 2012 and 2016, had a confirmed diagnosis of H1N1 infection, and met the clinical and biochemical criteria. Those criteria, as outlined by the U.S. Centers for Disease Control and Prevention^(^^6^^)^, included flu-like symptoms; a body temperature of 37.8°C or higher; cough or sore throat; rapid evolution; and positivity for the H1N1 virus on real-time reverse transcriptase polymerase chain reaction.

We included all patients who had undergone chest X-ray within the first 24 h after the initial clinical presentation and had a confirmed diagnosis of H1N1 infection. After reviewing the patient medical records for signs/symptoms, laboratory test results and imaging findings, as well as for the results of blood, urine, and sputum cultures (when available), we excluded the patients who had a confirmed diagnosis of another acute disease (e.g., bacteremia). Although 25 patients were initially selected, eight were excluded: five because examinations and data were missing from their medical records; and three because the diagnosis of H1N1 infection was not confirmed. Therefore, the final sample comprised 17 cases.

We collected patient data on the following: age; gender; referral source; ethnicity; history of vaccination against H1N1; comorbidities and risk factors; symptoms; initial physical examination findings; laboratory test results; imaging findings; treatment instituted; admission to the intensive care unit; need for mechanical ventilation; length of hospital stay; and death. We also recorded the time from symptom onset to hospital admission.

### Evaluation of chest X-rays

All X-rays were acquired with a digital system (Digital Diagnostic TH/VR, Axiom MDF, Siemens, Erlangen, Germany). The images were reviewed in consensus by two radiologists (with 8 and 20 years of experience in thoracic imaging, respectively), one second-year radiology resident, and one third-year radiology resident. At our facility, X-rays of pediatric patients were routinely obtained in an anteroposterior view and, if necessary, in a lateral view. The two reviewers were blinded to the clinical data and clinical outcomes of the patients. Of the 17 patients in whom chest X-rays were obtained at admission, no previous imaging was available for comparison. The images were analyzed on a RIS/PACS workstation using a DicomViewer (Medical Connection Ltd., UK).

Each X-ray was first categorized as either normal or abnormal. The X-rays showing alterations (100% of the cases) were then analyzed as to the radiographic pattern. Findings that were considered likely to be related to the current infection with the influenza virus were then described in accordance with the radiographic pattern of involvement: peribronchovascular opacities; consolidation; nonspecific alveolar opacities; peribronchovascular opacities accompanied by consolidation; peribronchovascular opacities accompanied by nonspecific alveolar opacities; consolidation accompanied by nonspecific alveolar opacities; and peribronchovascular opacities accompanied by consolidation and nonspecific alveolar opacities. Consolidation was defined as a homogeneous increase in the attenuation of the lung parenchyma, obscuring the margins of the vessels and airways. A nonspecific alveolar opacity was defined as an area of increased, nebulous lung opacity, within which the definition of the pulmonary structures is usually preserved. Such an opacity is less opaque than is a consolidation and resembles the ground-glass pattern seen on CT scans. The images were interpreted using the description found in the Fleischner Society glossary^(^^7^^)^.

In each case, the distribution of the radiographic findings was categorized as either unilateral or bilateral. The findings were also categorized as either symmetric or asymmetric. In relation to the distance from the hilum, the findings were categorized as central (within 4 cm of the hilum), peripheral, or peribronchial. The extent of the findings was categorized as either less than 25% or greater than 25%^(^^8^^)^. In addition, the lungs were segmented into upper, middle, and lower zones by using imaginary horizontal lines dividing the lungs into those three levels, each zone representing one third of the long axis of each lung. The presence and size of pleural effusion were recorded, as were other factors, including the use of life support devices, increase in the cardiac silhouette, pulmonary edema, pneumothorax, and pneumomediastinum.

### Statistical analysis

The data were analyzed with the IBM SPSS Statistics software package, version 20.0 (IBM Corp., Armonk, NY, USA) and a Microsoft Excel 2010 spreadsheet. The data were entered in the Excel program and subsequently exported to the SPSS software for statistical analysis. Categorical variables were described as absolute and relative frequencies. Continuous variables with normal distribution were described as means and standard deviations, whereas those with abnormal distribution were described as medians and interquartile ranges (25th and 75th percentiles). Associations with categorical variables were determined by using Fisher's exact test. Variables with normal distribution were compared by using the Student's *t*-test for independent samples, whereas those with abnormal distribution were compared by using the Mann-Whitney test. In all comparisons, the level of significance was set at 5%.

## RESULTS

The mean age of the patients (10 females and 7 males) was 45.5 months. The duration of signs and symptoms ranged from 1 to 15 days. Eight patients (47.1%) presented no need for ventilatory support and nine (52.9%) needed such support after the initial evaluation in the department of emergency medicine, two of those nine evolving to death. In a survey concerning their vaccination status, we found that two (11.8%) of the patients had been vaccinated, four (23.5%) had not, and 11 (64.7%) had no record of it or provided no information. All of the patients presented with fever and cough. We also identified coryza in seven patients (41.2%), vomiting in six (35.3%), diarrhea in two (11.8%), and a sore throat in two (11.8%). The mean oxygen saturation was 93.5%, and the mean respiratory rate was 50.9 breaths per minute (Table 1).

**Table 1:** Demographic and clinical characteristics of pediatric patients with H1N1 infection.

Characteristic	Value (n = 17)
Female, n (%)	8 (47.1)
Age (months), median (interquartile range)	9 (52.9)
Not requiring ventilatory support, n (%)	10 (58.8)
Requiring ventilatory support, n (%)	14 (2-89)
Vaccination, n (%)	
Yes	2 (11.8)
No	4 (23.5)
No data	11 (64.7)
Death, n (%)	2 (11.8)
Oxygen saturation (%), mean ± SD	93.5 ± 9.9
Respiratory rate (breaths/min), mean ± SD	50.9 ± 18.1
Fever, n (%)	17 (100.0)
Cough, n (%)	17 (100.0)
Coryza, n (%)	7 (41.2)
Vomiting, n (%)	6 (35.3)
Diarrhea, n (%)*	2 (12.5)
Sore throat, n (%)	2 (11.8)
Duration of signs and symptoms (days), median	4 (1-15)
(interquartile range)	

* One patient was not assessed. SD, standard deviation.

**Figure 1:**
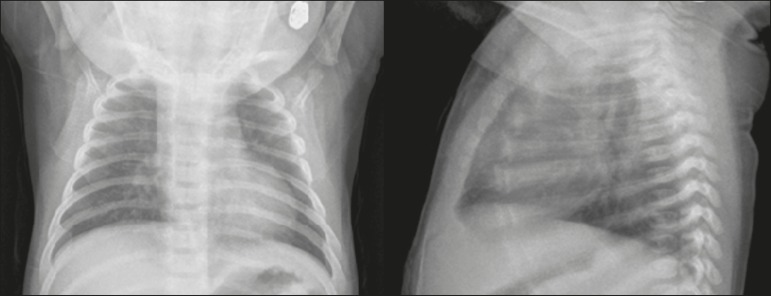
A 2-month-old female patient who presented with a 72-h history of productive cough and nasal obstruction, evolving to fever, dyspnea, and respiratory distress, therefore requiring oxygen therapy and continuous monitoring. An initial X-ray showing asymmetric peribronchovascular opacities in the middle third of the right lung, with central distribution and affecting less than 25% of the lung.

**Figure 2:**
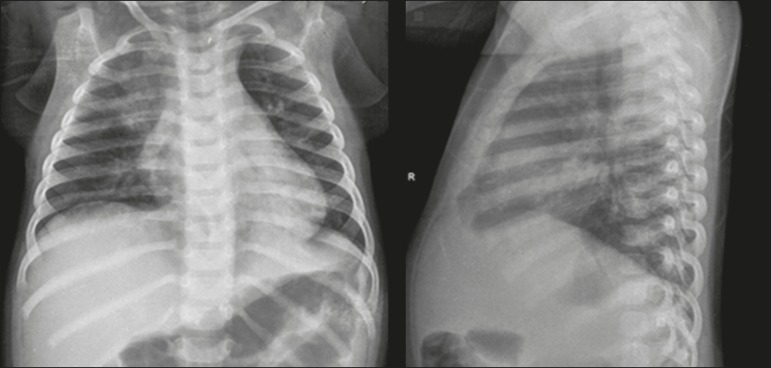
An 11-month-old female patient who presented with a 5-day history of fever, productive cough, and vomiting. An X-ray showing asymmetric unilateral alveolar consolidation in the upper third of the right lung, with central and peripheral distribution and an extent of less than 25%, together with a retrocardiac opacity on the left.

**Figure 3:**
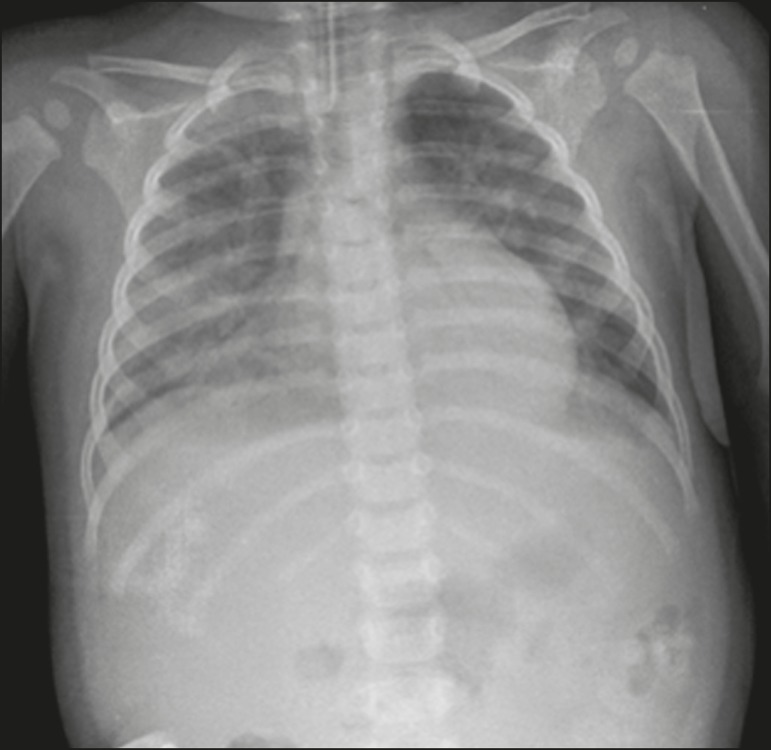
A 13-month-old female patient who presented with productive cough and coryza, evolving to loss of appetite, adynamia, and a decline in general health status. She evolved to drowsiness, intense dyspnea, and high fever, requiring ventilatory support. An X-ray showing asymmetric bilateral nonspecific alveolar opacities involving the entire lung, with central and peripheral distribution, affecting more than 25% of the lung.

The characteristic found most frequently on the X-rays (Table 2) was peribronchovascular opacity (Figure 1), which were seen in five patients (29.0%), and the extent of the involvement was less than 25% in nine patients (52.9%). Consolidation (Figure 2) was reported in three cases (17.6%), and nonspecific alveolar opacities (Figure 3) were reported in two (11.8%). Multiple or concomitant abnormalities were seen in seven patients (41.2%). The changes were bilateral in 11 (64.7%) of the examinations and asymmetric in 13 (76.5%). We observed involvement of the entire lung parenchyma in nine patients (52.9%), of the middle third in five (29.4%), of the upper third in one (5.9%), and of the lower third in two (11.8%). No peribronchovascular involvement was detected in any of the examinations evaluated. Pleural effusion was observed in four cases (23.5%), pneumothorax was observed in two (11.8%), and pneumomediastinum was observed in one (5.9%).

**Table 2:** Radiographic findings in pediatric patients with H1N1 infection.

Radiographic finding	Value (n = 17)
Abnormalities on initial X-ray, n (%)	
Peribronchovascular opacities	5 (29.4)
Nonspecific alveolar opacity	2 (11.8)
Peribronchovascular opacities + ground-glass pattern	4 (23.5)
Consolidation	3 (17.6)
Peribronchovascular opacities + consolidation	1 (5.9)
Consolidation + nonspecific alveolar opacity	1 (5.9)
Peribronchovascular opacities + nonspecific alveolar	1 (5.9)
opacity + consolidation	
Consolidation in isolation or accompanied by other	6 (35.3)
patterns, n (%)	
Extent, n (%)	
< 25%	9 (52.9)
> 25%	8 (47.1)
Laterality, n (%)	
Bilateral	11 (64.7)
Unilateral	6 (35.3)
Symmetry, n (%)	
Symmetric	4 (23.5)
Asymmetric	13 (76.5)
Zone involved, n (%)	
Upper	1 (5.9)
Middle	5 (29.4)
Lower	2 (11.8)
All	9 (52.9)
Distribution, n (%)	
Central and peripheral	10 (58.8)
Central	6 (35.3)
Peripheral	1 (5.9)
Pleural effusion, n (%)	
Unilateral	4 (23.5)
Absent	13 (76.5)

In comparing radiographic findings between patients with and without ventilatory support (Table 3), we detected a statistically significant difference between the groups in terms of symmetry: in patients on ventilatory support, there was a preponderance of asymmetric findings, whereas in patients not on ventilatory support, findings were equally distributed between symmetric and asymmetric (*p* = 0.029). Other aspects, such as consolidations (either in isolation or accompanied by other patterns), the extent of the lesions, laterality, symmetry, the zone involved, and distribution, were not statistically significant in this context, nor were they in the comparison of the clinical characteristics of patients requiring and not requiring ventilatory support (Table 4).

**Table 3:** Radiographic findings in pediatric patients with H1N1 infection, according to the need for ventilatory support.

Radiographic finding	Ventilatory support required (n = 8)	Ventilatory support not required (n = 9)	P
Abnormalities on initial X-ray, n (%)			0.073
Peribronchovascular opacities	5 (62.5)	-	
Nonspecific alveolar opacity	1 (12.5)	1 (11.1)	
Peribronchovascular opacities + ground-glass pattern	1 (12.5)	3 (33.3)	
Consolidation	1 (12.5)	2 (22.2)	
Peribronchovascular opacities + consolidation	-	1 (11.1)	
Consolidation + nonspecific alveolar opacity	-	1 (11.1)	
Peribronchovascular opacities + nonspecific alveolar opacity + consolidation	-	1 (11.1)	
Consolidation in isolation or accompanied by other patterns, n (%)	1 (12.5)	5 (55.6)	0.131
Extent, n (%)			0.153
< 25%	6 (75.0)	3 (33.3)	
> 25%	2 (25.0)	6 (66.7)	
Laterality, n (%)			0.999
Bilateral	5 (62.5)	6 (66.7)	
Unilateral	3 (37.5)	3 (33.3)	
Symmetry, n (%)			0.029
Symmetric	4 (50.0)	-	
Asymmetric	4 (50.0)	9 (100.0)	
Zone involved, n (%)			0.361
Upper	-	1 (11.1)	
Middle	4 (50.0)	1 (11.1)	
Lower	1 (12.5)	1 (11.1)	
All	3 (37.5)	6 (66.7)	
Distribution, n (%)			0.079
Central and peripheral	3 (37.5)	7 (77.8)	
Central	5 (62.5)	1 (11.1)	
Peripheral	-	1 (11.1)	

**Table 4:** Clinical characteristics of pediatric patients with H1N1 infection, according to the need for ventilatory support.

	Ventilatory support required (n = 8)	Ventilatory support not required (n = 9)	*P*
Characteristic			
Female, n (%)	5 (62.5)	5 (55.6)	0.999
Age (months), median (interquartile range)	18 (2-72)	14 (9-89)	0.481
Vaccination, n (%)			0.999
Yes	1 (12.5)	1 (11.1)	
No	2 (25.0)	2 (22.2)	
No data	5 (62.5)	6 (66.7)	
Death, n (%)	-	2 (22.2)	0.471
Oxygen saturation (%), mean ± SD	93.4 ± 4.3	93.6 ± 13.4	0.971
Respiratory rate (breaths/min), mean ± SD	53.1 ± 24.6	48.6 ± 9.2	0.640
Fever, n (%)	8 (100.0)	9 (100.0)	1.000
Cough, n (%)	8 (100.0)	9 (100.0)	1.000
Coryza, n (%)	3 (37.5)	4 (44.4)	0.999
Vomiting, n (%)	1 (12.5)	5 (55.6)	0.131
Diarrhea, n (%)†	1 (14.3)	1 (11.1)	0.999
Sore throat, n (%)	-	2 (22.2)	0.471
Duration of signs and symptoms (days), median (interquartile range)	4 (1-7)	4 (1-15)	0.481

* One patient was not assessed. SD, standard deviation.

## DISCUSSION

Most recent articles about thoracic radiology in infectious diseases address aspects of CT^(^^8^^-^^11^^)^. However, chest X-ray still plays an important role, especially in pediatric patients^(^^12^^)^. There have been few studies evaluating the initial chest X-ray findings in pediatric patients suspected of being infected with the H1N1 virus. The difficulties in differentiating between viral pneumonia and other infectious processes, such as bronchiolitis, on the basis of radiographic findings, especially in patients under two years of age, are well known. The cause of infection (viral vs. bacterial or fungal) cannot be reliably determined from the imaging characteristics^(^^13^^)^. However, pediatricians have an expectation that the findings of X-ray examinations, in correlation with the clinical data and laboratory test results, can guide the management of patients with H1N1 infection.

Regarding the clinical characteristics listed, our study showed that there was no statistical difference in the clinical criteria that distinguished the group that required ventilatory support from the group that did not, in which the outcomes were better. Peribronchovascular opacities, with or without other patterns, were identified as the most prevalent radiographic finding, occurring in 64.7% of the population studied. Nonspecific alveolar opacities were the second most common finding. Our findings regarding the prevalence of those abnormalities were similar to those of other authors^(^^14^^)^. However, the frequency of abnormal radiographic findings in our patients was different from that reported in studies of adult patients, one such study having reported a frequency of only 27%^(^^15^^)^, compared with 100% in our sample.

The radiographic pattern of peribronchovascular opacities was similar to that observed in infection with respiratory syncytial virus and infection with the parainfluenza virus, both of which are commonly seen in pediatric patients^(^^16^^)^. Although the reason for the absence of that finding among adult patients is uncertain, it could be related to immunological differences between the pediatric and adult populations. For example, it is possible that pediatric patients who have not yet developed substantial immunity to a number of viruses that infect the lower respiratory tract may have a mild, self-limited form of H1N1 infection visible on chest X-rays, similar to how they respond to viruses such as respiratory syncytial virus and the parainfluenza virus. In contrast, adult patients may not show such radiographic abnormalities if they have already been exposed to several respiratory tract viruses in their childhood^(^^16^^)^. As a result, adult patients may have a more robust immunity to new viral infections.

Our results suggest that chest X-rays play an important role in helping diagnose, treat, and perform appropriate follow-up of patients with a laboratory-confirmed diagnosis of H1N1 influenza infection. The asymmetry of lung involvement showed statistical significance to distinguish the group with a worse prognosis, given that the patients who required mechanical ventilation either died or had a preponderance of asymmetric findings.

The findings described in previous influenza pandemics have shown that radiographic manifestations of an influenza infection include segmental consolidation, which may be smooth or irregular and unilateral or bilateral. Our report corroborates those findings, because consolidation was common, being found, either in isolation or accompanied by other patterns, in 35% of the patients. Serial X-rays can show irregular areas of consolidation, 1-2 cm in diameter, which rapidly become confluent^(^^17^^,^^18^^)^. In one previous study of influenza infection, the number of cases of unilateral involvement was found to be approximately the same as that of cases of bilateral involvement and the disease was widely disseminated in approximately one-fourth of the patients with bilateral opacities, whereas pleural effusion was rare^(^^19^^)^.

Histopathological studies have shown that the acute stage of influenza infection is characterized by multifocal destruction, with desquamation of the epithelium (tracheal and bronchial), marked pulmonary edema, and congestion of the submucosa^(^^20^^)^. There can be necrosis in the bronchioles, together with peribronchial hemorrhage and peribronchial pneumonia. Severe infection is characterized by the following features^(^^20^^)^: necrotizing bronchitis; thrombosis of the capillaries and small blood vessels; interstitial edema and inflammatory infiltrates; the formation of hyaline membranes; alveolar hemorrhage and edema; and diffuse alveolar damage. Other authors have correlated the CT findings of consolidation with alveoli filled with exudate-either inflammatory or hemorrhagic. Those authors found that the ground-glass pattern reflected thickening of the interlobular septa due to inflammation or edema, with or without partial filling of the airspace^(^^21^^)^. Nevertheless, additional studies are needed in order to improve understanding of the pathophysiological basis of the findings, as well as of the ways in which the H1N1 virus interacts with the pulmonary endothelium and airway epithelium. From a practical standpoint, radiologists and clinicians should be aware that prominent peribronchovascular opacities may be associated with influenza virus infection in pediatric patients.

This study has several limitations. First, our study sample was relatively small, comprising only 17 patients. In addition, we evaluated only X-ray findings. Only one patient underwent a CT scan of the chest, and those images were not available at the time of the radiographic evaluation. Furthermore, because the study was conducted at a referral hospital, there might have been a selection bias, given that the cases referred to our institution are those presenting a risk factor for a worse prognosis or those with a moderate to severe clinical presentation. Moreover, we used a consensus reading rather than assessing interobserver agreement, which could be considered a disadvantage in the interpretation of chest X-rays^(^^22^^)^. Yet another limitation was that the radiographic findings were not confirmed by CT of the chest, which can show subtle changes in the lung not detected by conventional X-ray^(^^23^^)^.

## CONCLUSION

The chest X-ray findings in pediatric patients with H1N1 infection include peribronchovascular opacities, nonspecific alveolar opacities, and pulmonary consolidation, as well as other patterns. Asymmetry of the pulmonary involvement showed statistical significance in distinguishing a group that evolved to a worse outcome-the patients requiring mechanical ventilation-who either died or had a preponderance of asymmetric findings. The definitive diagnosis is made by correlating the X-ray findings with the clinical symptoms and the laboratory test results.
